# Research status and development trend of extracorporeal membrane oxygenation based on bibliometrics

**DOI:** 10.3389/fcvm.2023.1048903

**Published:** 2023-03-10

**Authors:** Cuizhong Guan, Hua Shen, Shiyong Dong, Yuhua Zhan, Jie Yang, Qiu Zhang, Rong Wang

**Affiliations:** ^1^ Tsinghua University Library, Tsinghua University, Beijing, China; ^2^ Department of Adult Cardiac Surgery, The Sixth Medical Centre of PLA General Hospital, Beijing, China; ^3^ Department of Cardiovascular Surgery, The First Medical Centre of PLA General Hospital, Beijing, China

**Keywords:** extracorporeal membrane oxygenation, COVID-19, bibliometrics, citespace, hotspots, R&D centers, medicine enterprises

## Abstract

**Background:**

Using bibliometric method to analyze the research status and development trend of extracorporeal membrane oxygenation (ECMO), we aim to provide clinicians, scientists, and stakeholders with the most up-to-date and comprehensive overview of ECMO research.

**Materials and methods:**

Using Excel and VOSviewer, the literature on ECMO was systematically analyzed regarding publication trends, journal source, foundation, countries, institutions, core authors, research hotspots, and market distribution.

**Results:**

There were five important time nodes in the research process of ECMO, including the success of the first ECMO operation, the establishment of ELSO, and the outbreak of influenza A/H1N1 and COVID-19. The R&D centers of ECMO were the United States, Germany, Japan, and Italy, and the attention to ECMO was gradually increasing in China. The products most used in the literature were from Maquet, Medtronic, and LivaNova. Medicine enterprises attached great importance to the funding of ECMO research. In recent years, the literature has mainly focused on the following aspects: the treatment of ARDS, the prevention of coagulation system-related complications, the application in neonatal and pediatric patients, mechanical circulatory support for cardiogenic shock, and ECPR and ECMO during the COVID-19 pandemic.

**Conclusion:**

The frequent epidemic occurrence of viral pneumonia and the technical advancement of ECMO in recent years have caused an increase in clinical applications. The hot spots of ECMO research are shown in the treatment of ARDS, mechanical circulatory support for cardiogenic shock, and the application during the COVID-19 pandemic.

## 1. Introduction

The coronavirus disease 2019 (COVID-19) caused by severe acute respiratory syndrome coronavirus 2 (SARS-CoV-2) is now rampantly spreading around the world (1). According to data released by World Health Organization (WHO), the cumulative worldwide number of confirmed cases of new COVID reached 754 million, and that of deaths reached more than 6.82 million as of February 4, 2023. Although patients have mild symptoms in most cases, a certain percentage of patients develop severe respiratory and circulatory failure requiring admission to the intensive care unit. Many patients receive invasive mechanical ventilation, but the mortality rate is extremely high (2). Extracorporeal membrane oxygenation (ECMO) has become the “ultimate weapon” to save critically ill patients (3), and the WHO has included ECMO in the treatment protocol for pneumonia in novel coronavirus infections (4).

Despite the growing interest in ECMO among clinicians and scientists, there are no comprehensive studies looking at global research activity in ECMO due to the large number and heterogeneity of ECMO-related scientific publications, making it difficult for individual researchers to gauge their individual scholarly value by reading all published publications and keeping abreast of the latest research advances.

Based on the ECMO-related scientific publications in the Science Citation Index Expanded (SCIE) database (5), this study used scientometric methods to analyze global research productivity, international collaborations, research themes. Combined with visualization software, we provided an overview of global research activities, international collaborative network structures, and recent research hotspots since the birth of ECMO in the 1950 s, with the aim of providing clinicians, scientists, and stakeholders with the most up-to-date and comprehensive overview of ECMO research.

## 2. Methods

This study used the Science Citation Index Expanded (SCIE) database as the source of data collection, and the data were searched in the time range up to December 31, 2022. The search time was January 25, 2023. All papers related to ECMO research were searched by title, keywords and topic, and the valid papers included 9,082 papers from 82 countries/regions after refining the document type as “article”. Review articles are not included in this analysis to avoid slanting the results in one direction or another.

**Figure 1:**
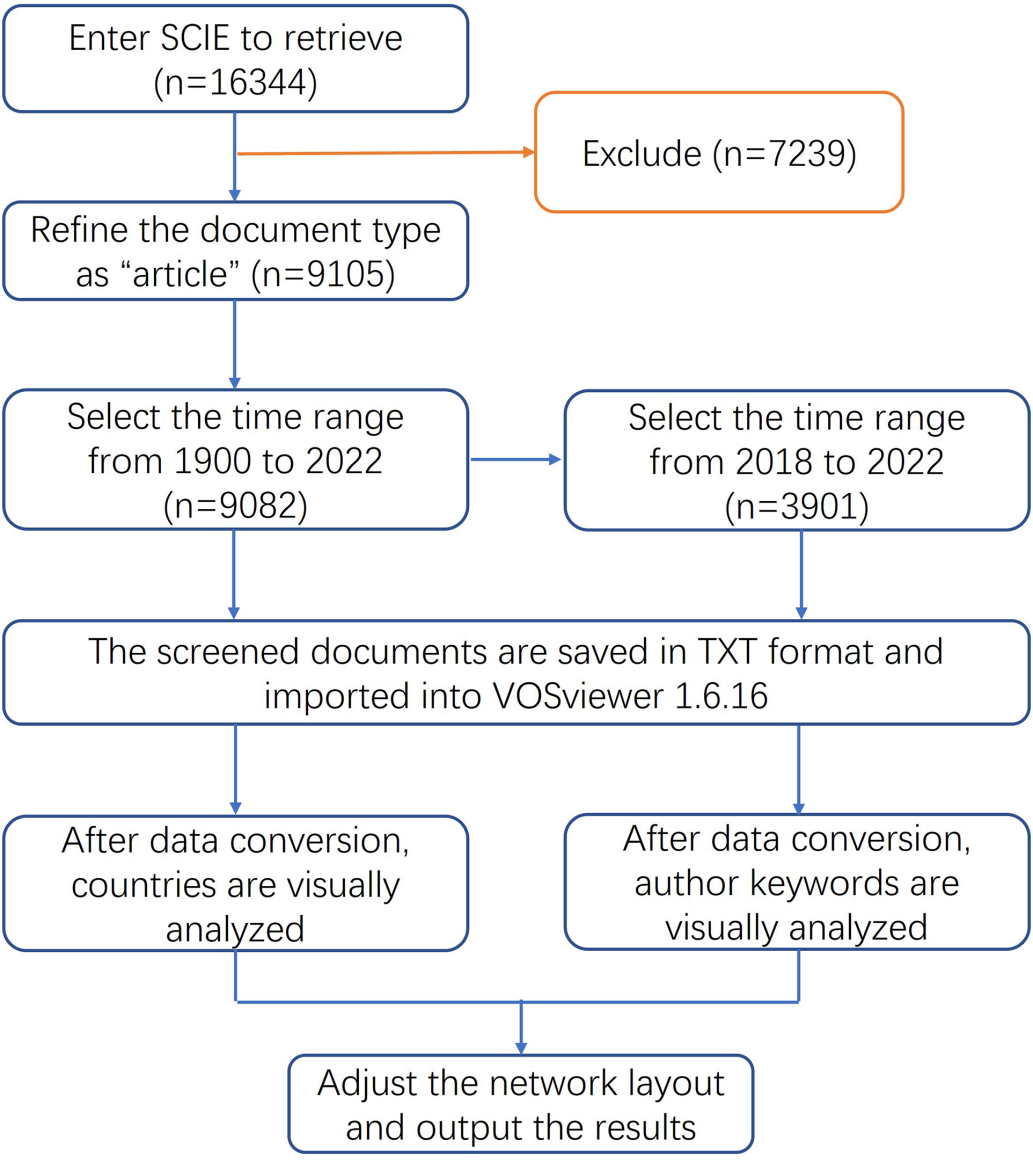
Visual analysis flow chart of ECMO research field.

After the literature search, this paper used Excel 2019 and VOSviewer 1.6.16 to statistically analyze the data (6), explore the literature growth trends and stage distribution characteristics, analyze the journals and funding sources of papers, and study the status of countries/institutions and author groups. With the help of VOSviewer developed by the Centre for Science and Technology Studies (CWTS) at Leiden University in the Netherlands, this study analyzed the countries and author keywords of the literature and summarized the cooperation network and hot spots of ECMO research in recent years (Figure 1). Finally, a statistical analysis of the use of the products from major ECMO suppliers in the relevant research papers was performed.

## 3. Manuscript formatting

### 3.1. Overview of the study

#### 3.1.1. Analysis of paper output

**Figure 2:**
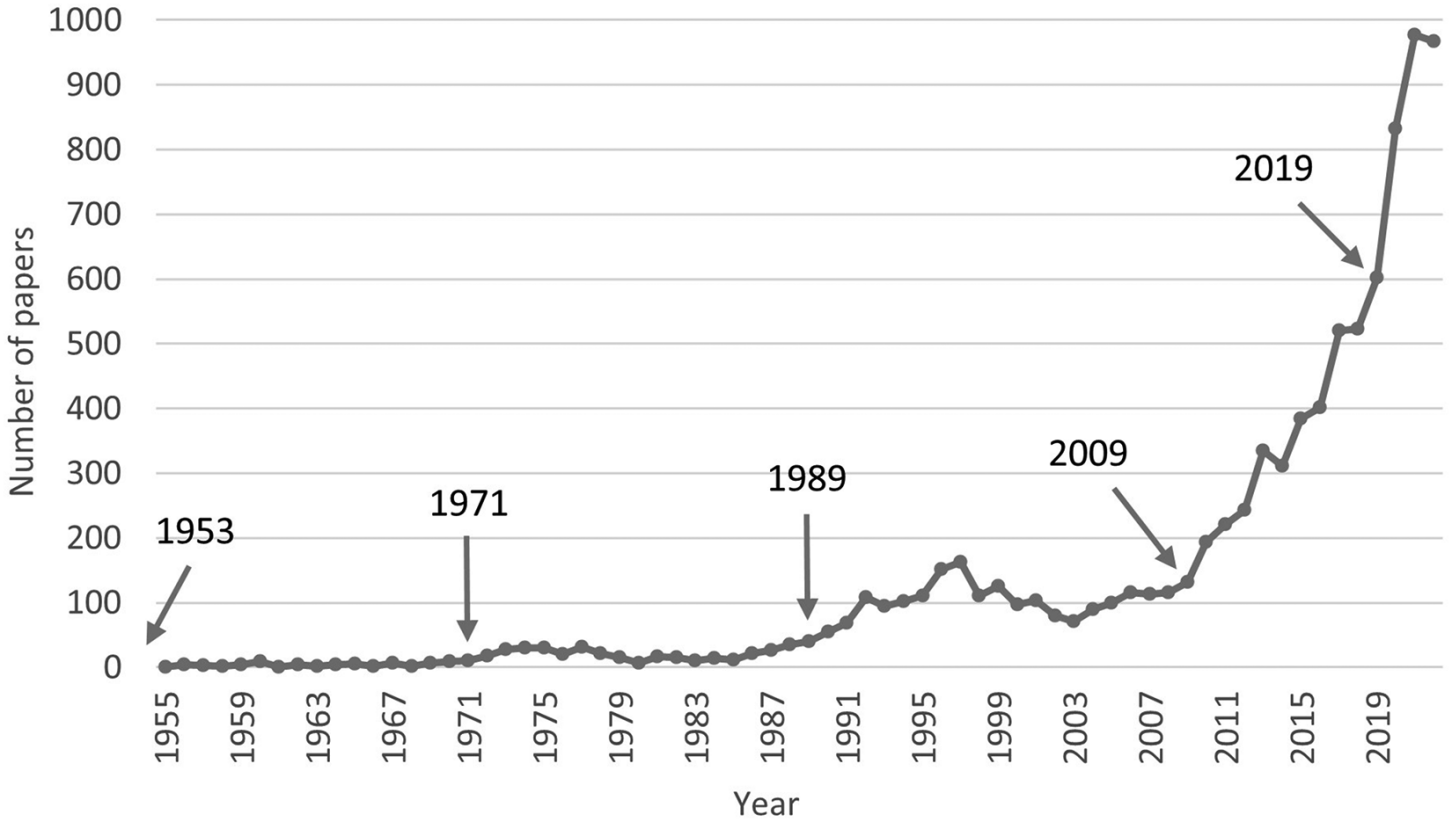
Chronological distribution of ECMO research papers.

The number of academic papers is closely related to the application demand and technical level of ECMO (7). The frequent epidemic occurrence of viral pneumonia and the technical advancement of ECMO in recent years have caused an increase in clinical applications and improved treatment outcomes, while the gradual accumulation of successful clinical applications has, in turn, promoted the technical advancement of ECMO. A review of the number of ECMO papers over the years revealed that there were five important time points in the research history (Figure 2).

1953: The father of extracorporeal circulation, John Heysham Gibbon Jr, first applied the use of extracorporeal circulation to perform cardiac surgery with success in 1953 (8). ECMO was actually an extension and prolonged application of conventional extracorporeal circulation techniques. Since then, the research on extracorporeal circulation technology has entered a slow development phase, exploring the field of oxygenator and blood anticoagulation technology development, with less than 10 papers per year.

1971: In 1971, Dr. Hill first used ECMO to save an adult male patient with progressive respiratory failure due to multiple traumas. After 75 h of ECMO treatment, the patient was successfully saved. In this regard, a review article “Buying time with artificial lungs” was published in the New England Journal of Medicine (NEJM) (9). Additionally, Dr. Bartlett first successfully used ECMO in the treatment of severe respiratory failure in neonates in 1975 and led the establishment of the world first ECMO center in 1980 (10).

1989: As the number of patients successfully treated by ECMO increased, the Extracorporeal Life Support Organization (ELSO) was established in 1989 to provide support to those delivering extracorporeal life support through continuing education, guidelines, original research, publications, and a comprehensive registry of extracorporeal membrane oxygenation (ECMO) patient data (11). ECMO technology was promoted to a certain extent in the 1990 s as its clinical applications increased. The number of papers increased more significantly, reaching 100 papers per year. However, the early clinical studies did not show that ECMO improved the prognosis of acute respiratory distress syndrome (ARDS) patients due to the shortcomings and deficiencies of the timing of ECMO initiation and significant adverse events, such as severe bleeding. This limited the development of ECMO with a small drop in the number of related papers after 2000.

2009: Influenza A (H1N1) began a massive global pandemic in 2009, and ECMO was successfully applied in the treatment of ARDS patients with influenza A (H1N1) 2009 infection (12, 13). In the same year, the results of the CESAR trial showed that the incidence of the primary composite endpoint (death or severe disability at 6 months) was significantly lower in the ECMO group than in the conventional treatment group (14). The use of ECMO technology for the adjuvant treatment of adult ARDS rapidly increased. With the increased clinical experience in applying ECMO and the gradual improvement of key technologies, the clinical efficacy of ECMO support significantly improved (15). The number of related studies rapidly increased to reach 602 papers in 2019.

2019: Since the new coronavirus disease outbreak in late 2019, ECMO has become a famous tool to fight against the epidemic (3, 4). ECMO application was included in the “Clinical Management of COVID-19” by the WHO, which recommended the provision of ECMO for eligible patients with COVID-19-related ARDS. From 2020 to 2022, the number of ECMO-related papers significantly increased to nearly 1,000 per year.

#### 3.1.2. Distribution of journals

**Figure 3:**
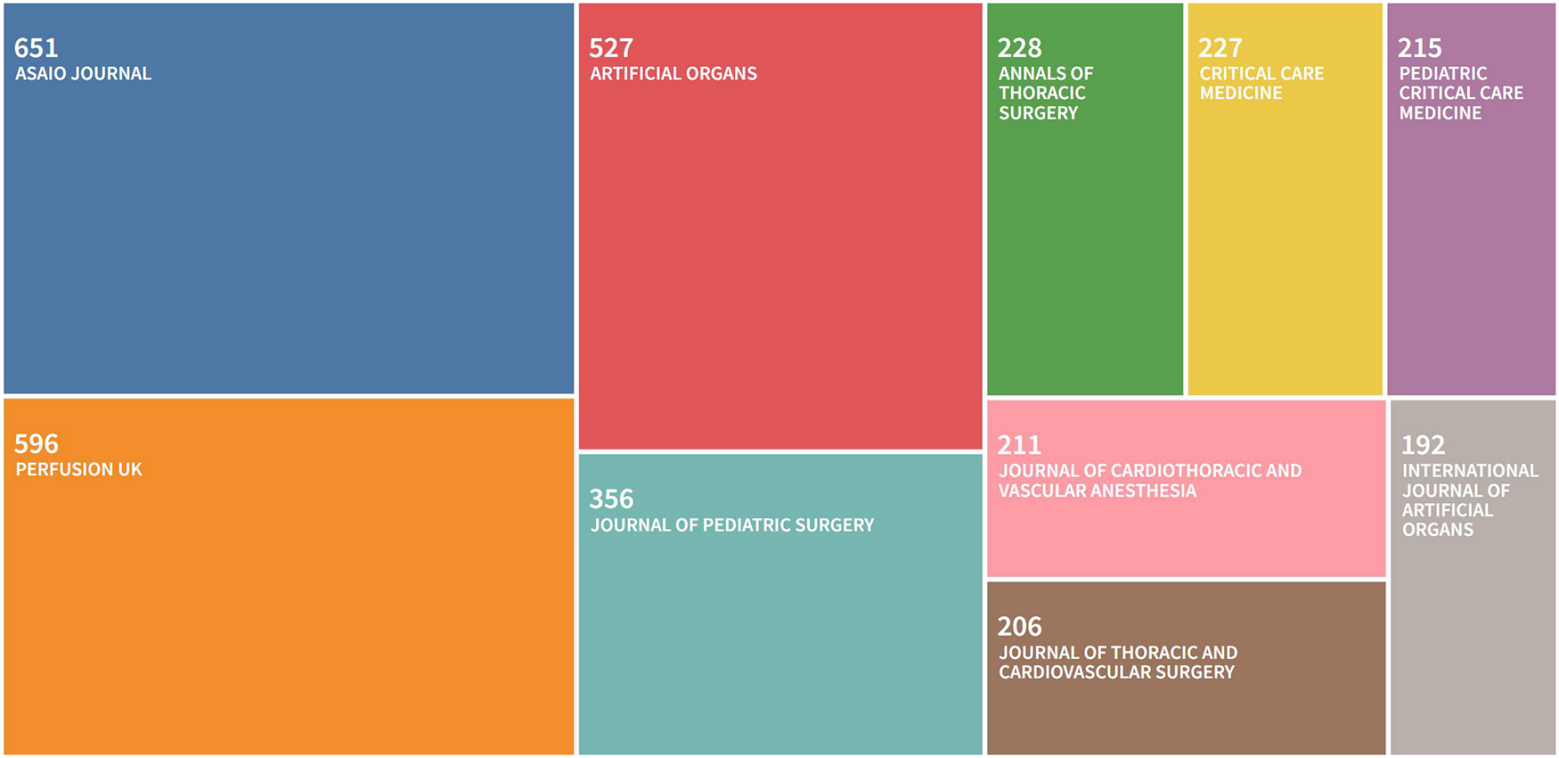
Top 10 source journals for ECMO research papers.

Papers on ECMO were distributed in 1,008 journals, of which 697 journals published less than three papers, accounting for about 69.1% of the total number of journals and carrying only 11.3% of the total literature. The main papers were concentrated in few journals (Figure 3), with 23 journals (2.3%) containing 50% of relevant papers and 159 journals (15.8%) containing 80% of relevant papers, in line with the 80/20 rule of journals (16). The journal with the highest number of articles was ASAIO JOURNAL, which published 651 ECMO-related papers (accounting for 7.2%), followed by PERFUSION UK (596 papers, accounting for 6.6%) and ARTIFICIAL ORGANS (527 papers, accounting for 5.8%).

#### 3.1.3. Funding support

The United States Department of Health Human Services funded 555 papers (17, 18), far ahead of the other agencies and five times more than the second place National Natural Science Foundation of China (19) (Table 1). Interestingly, pharmaceutical companies also placed great emphasis on funding research at universities and hospitals, with Maquet (20) and Pfizer (21) ranking fourth and ninth in the top 10 funding agencies.

**Table 1:** Top 10 funding sources for ECMO research papers.

No.	Funding agency	Number of papers
1	HHS (United States Department of Health Human Services)	555
2	NSFC (National Natural Science Foundation of China)	93
3	DFG (German Research Foundation)	66
4	Maquet	54
5	Projekt DEAL	51
6	NHMRC (National Health and Medical Research Council of Australia)	49
7	European Commission	47
8	MEXT (Ministry of Education Culture Sports Science And Technology Japan)	39
9	Pfizer	31
10	UKRI (UK Research Innovation)	30

#### 3.1.4. Country/region distribution

**Figure 4:**
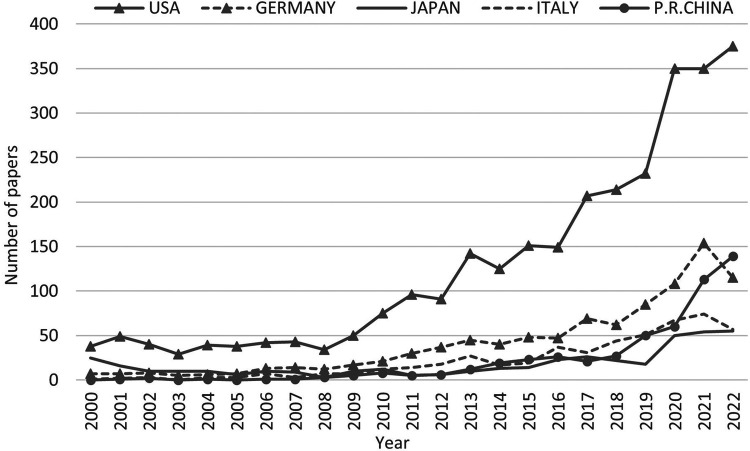
Trend of ECMO research papers published in selected countries/regions since 2000.

There are 82 countries/regions involved in ECMO research worldwide, which is widely distributed, although the main research strength is concentrated in the United States, Europe, and Japan (Table 2). The United States is far ahead of other countries/regions in the field of ECMO research, with 3,806 papers, accounting for 41.9% of the total literature, and is the center of global ECMO research. Germany is in second place, with 1,042 papers, accounting for 11.5% of the total literature, followed by Japan, Italy, P.R.China. The United States, Germany, and Italy are home to the top three ECMO brands in the world: Medtronic, Maquet, and Sorin, respectively. P.R.China ranked fifth, with 524 total papers, and the number of papers before 2013 was <10 papers per year, which developed faster in recent years. The number of papers published in P.R.China reached 139 in 2022, second only to the United States, showing that the importance and research strength of ECMO research in P.R.China has reached an international advanced level (Figure 4).

**Table 2:** Distribution of Top 10 source countries/regions of ECMO research papers.

Country/Region	Number of papers	Percentage	Country/Region	Number of papers	Percentage
United States	3,806	41.9%	France	501	5.5%
Germany	1,042	11.5%	England	465	5.1%
Japan	588	6.5%	Australia	383	4.2%
Italy	525	5.8%	Canada	336	3.7%
P.R.China	524	5.8%	South Korea	307	3.4%

**Figure 5:**
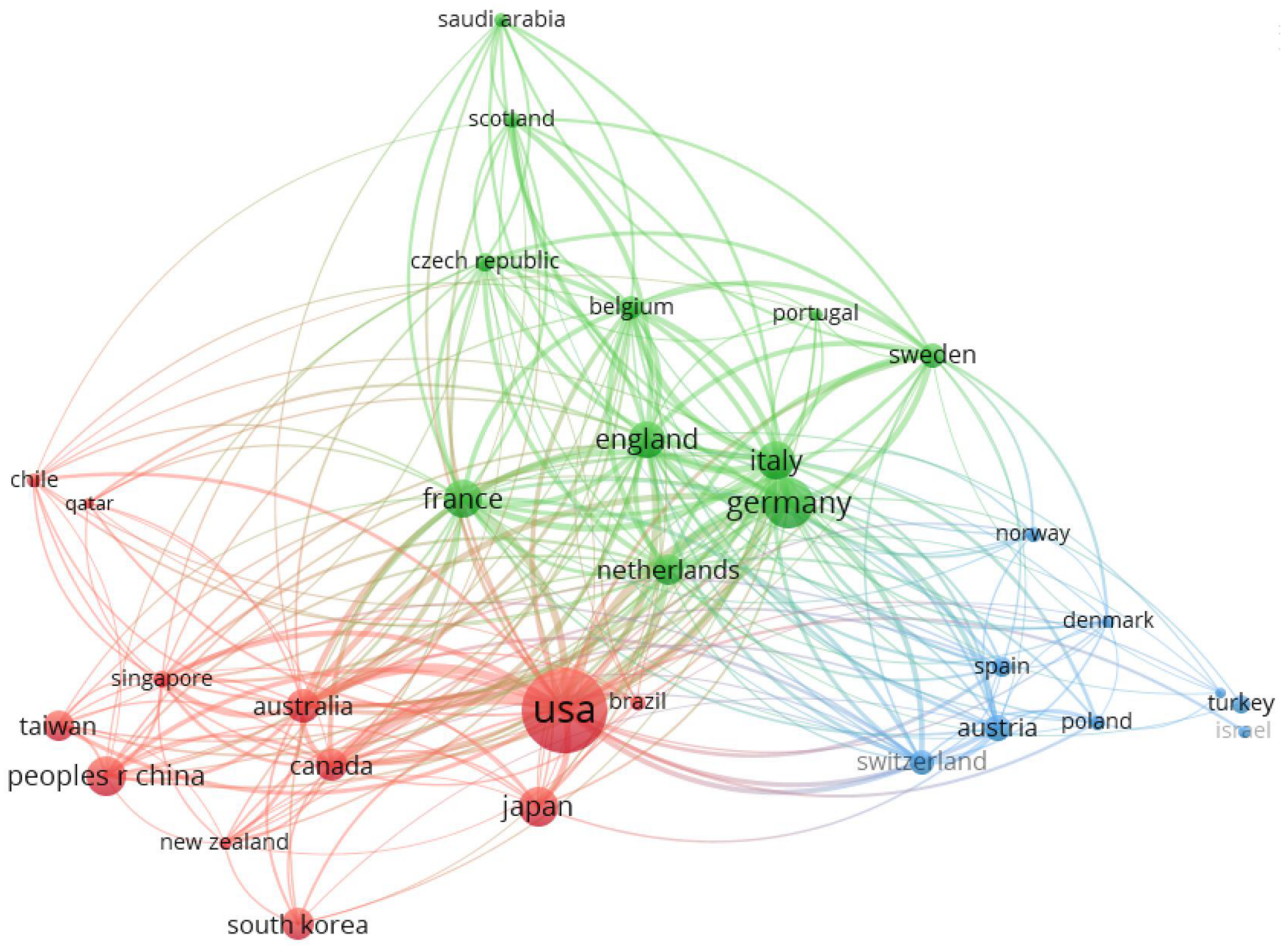
Cooperation network in the field of ECMO research.

Using VOSviewer, a visual network graph of international collaboration between countries/regions was drawn based on ECMO research papers (Figure 5). Each bubble represents a country/region. The bubble size represents the number of papers in that country/region. The connecting line between the bubbles represents the papers published in collaboration between two countries/regions. The thickness of the line indicates the number of papers in international collaboration, and the clustering to the same color indicates closer collaboration between related countries/regions. As can be seen from the figure, the international cooperation among countries/regions worldwide is mainly divided into the following three clusters.

(1) Red: centered in the United States, Japan, P.R.China, Australia, Canada, South Korea.
(2) Green: centered in the Germany, Italy, France, England, Netherlands.
(3) Blue: centered in Austria, Switzerland, Spain, Turkey.

#### 3.1.5. Affiliations/authors analysis

All 9,082 papers on ECMO were analyzed according to their affiliations and authors. The 20 most productive ECMO research institutions in the world were mainly located in the United States, France, England (Table 3). Among them, ten were in the United States, including the University of Michigan, Harvard University, and Columbia University. Four were in France, namely the Assistance Publique Hopitaux Paris, Institut National de la Sante et de la Recherche Medicale, Sorbonne Universite, and Hopital Universitaire Pitie Salpetriere APHP. Two were in England, namely the University of Leicester and the University of London. The most productive author of ECMO was Bartlett Robert H. of the University of Michigan (10). Dr. Bartlett is known around the world as the father of ECMO and he has published 144 papers in the field of ECMO. He was followed by Professor Brodie Daniel (22) of Columbia University (103 articles) and Professor Philipp Alois (23) of University of Regensburg (99 articles). Bartlett Robert H. and Brodie Daniel were all involved in writing the latest ELSO guidelines: “Extracorporeal Membrane Oxygenation for COVID-19: Updated 2021 Guidelines from the Extracorporeal Life Support Organization” (24).

**Table 3:** Top 20 institutions in terms of number of ECMO research papers.

No.	Institution	Country/Region	Number of papers
1	University of Michigan	United States	313
2	Harvard University	United States	309
3	Assistance Publique Hopitaux Paris	France	272
4	Columbia University	United States	242
5	University of Pennsylvania	United States	212
6	Baylor College of Medicine	United States	197
7	Institut National de la Sante et de la Recherche Medicale	France	188
8	University of Pittsburgh	United States	183
9	Boston Children's Hospital	United States	183
10	New York Presbyterian Hospital	United States	178
11	Sorbonne Universite	France	170
12	University of Regensburg	Germany	155
13	University of Toronto	Canada	150
14	Johns Hopkins University	United States	148
15	Hopital Universitaire Pitie Salpetriere APHP	France	145
16	Karolinska Institutet	Sweden	136
17	University of Maryland Baltimore	United States	136
18	University of Leicester	England	125
19	University of London	England	123
20	Maastricht University	Netherlands	102

An analysis of the major research institutions and authors in the field of ECMO in P.R.China showed that Capital Medical University had the highest productivity (76 articles), followed by the Chinese Academy of Medical Sciences & Peking Union Medical College (55 articles) and Zhejiang University (51 articles). The most productive authors were, in order, Hou Xiaotong (25), Wang Hong (26) and Wang Liangshan from Beijing Anzhen Hospital, Capital Medical University.

### 3.2. Analysis of research hotspots

**Figure 6:**
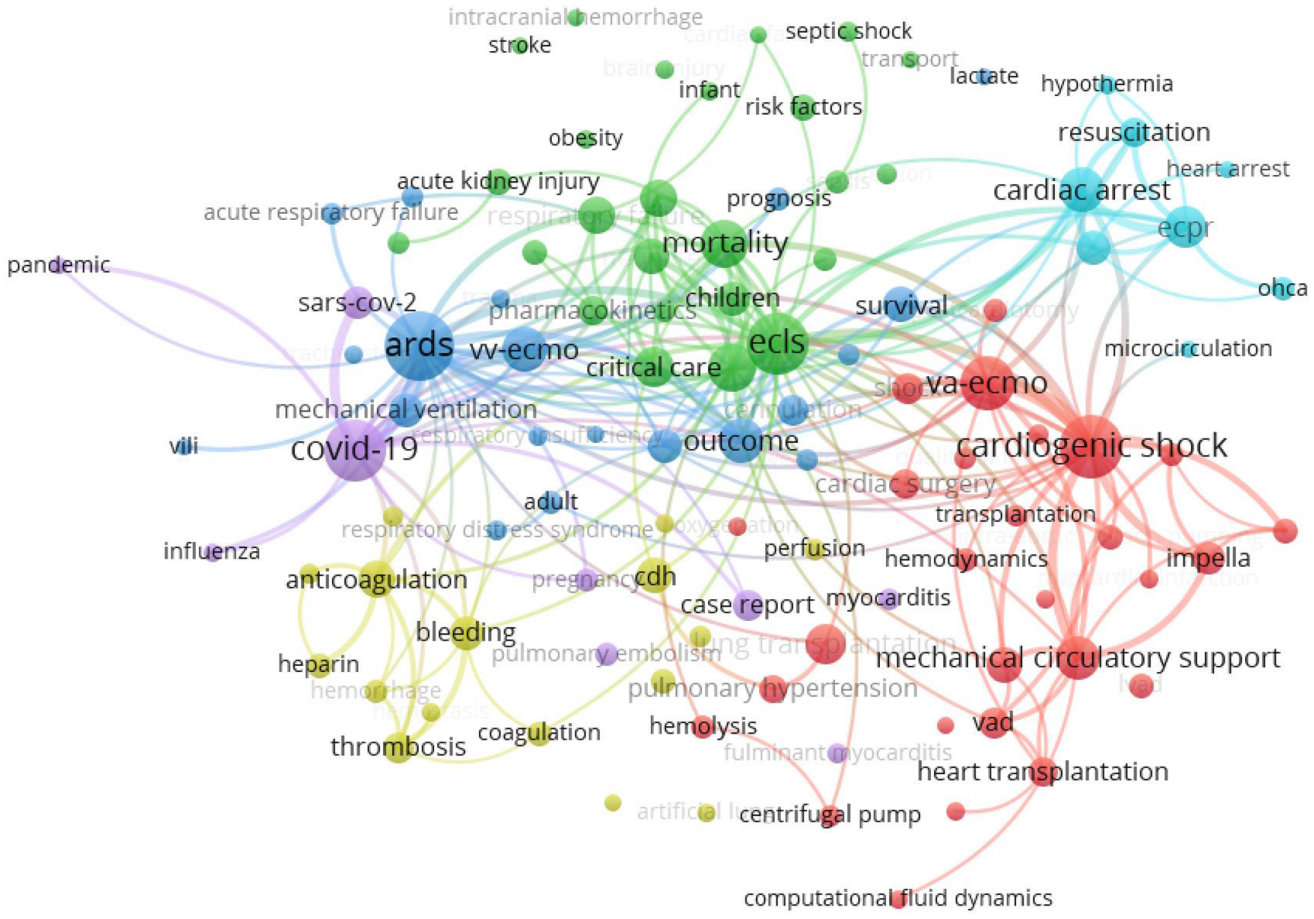
Keyword co-occurrence mapping of ECMO research papers, 2018–2022.

The keyword analysis of the papers in recent years can provide a quick understanding of the current research hotspots in the field. Here, the keyword co-occurrence clustering analysis of relevant research papers from 2018 to 2022 was performed using the VOSviewer visualization tool to build the knowledge map of the global ECMO research field, understand the international development and application status of the field as a whole, and reveal the research hotspots and frontiers of ECMO (Figure 6). The research hotspots were classified into the following six clusters based on keywords in the literature.

Red cluster 1 focused on the use of VA ECMO in cardiogenic shock, such as acute myocardial infarction, and acute heart failure, as well as in mechanical circulatory support, such as ventricular assist devices and bridging to heart transplantation (27). Additionally, ECMO also plays a key role in critical care support and surgical treatment for lung transplantation, heart transplantation and pulmonary hypertension.

Green cluster 2 focused on the application of ECMO in the critical care field in neonatal care and pediatrics (28), in which the research on ECMO support for acute kidney injury and congenital heart disease was among the hot spots.

Blue cluster 3 focused on the use of VV ECMO in ARDS, the most widely used area of ECMO clinical application, with several large clinical studies confirming its key role in respiratory support and improved prognosis in ARDS (29).

Yellow cluster 4 mainly included the study of coagulation system-related complications, such as anticoagulation or bleeding (30, 31). This has always been the focus and difficulty of ECMO research regarding the question of how to optimize anticoagulation protocols and improve anticoagulation coating techniques to reduce the risk of bleeding and thromboembolic.

Sky blue cluster 5 suggested that another research hotspot of ECMO focused on extracorporeal cardiopulmonary resuscitation (ECPR) (32). The indications for ECPR include in-hospital or out-of-hospital cardiac arrest due to acute hypothermia, acute cardiac infarction, and other causes. However, the choice of ECPR is still limited by the complexity of the causes of out-of-hospital cardiac arrest, the timing of resuscitation, neurological complications, and ethical issues.

Purple cluster 6 indicated the role of ECMO in the fight against the COVID-19 epidemic (3, 4). The recommendation for its application in the treatment of critically ill patients was included in the “Clinical Management of COVID-19” by the WHO. However, it should be noted that ECMO was also included as a restricted technology in the National Catalogue of Restricted Technologies (2022 version) and the National Management Specification for Clinical Application of Restricted Technologies (2022 version) issued by the National Health Care Commission of the PRC, considering its high technical threshold, strict assessment of medical institutions and medical personnel, and serious complications associated with it. The inclusion of ECMO as a restricted technique further highlights the need for research on ECMO indications, contraindications, and the reduction of complications.

### 3.3. Market distribution

**Figure 7:**
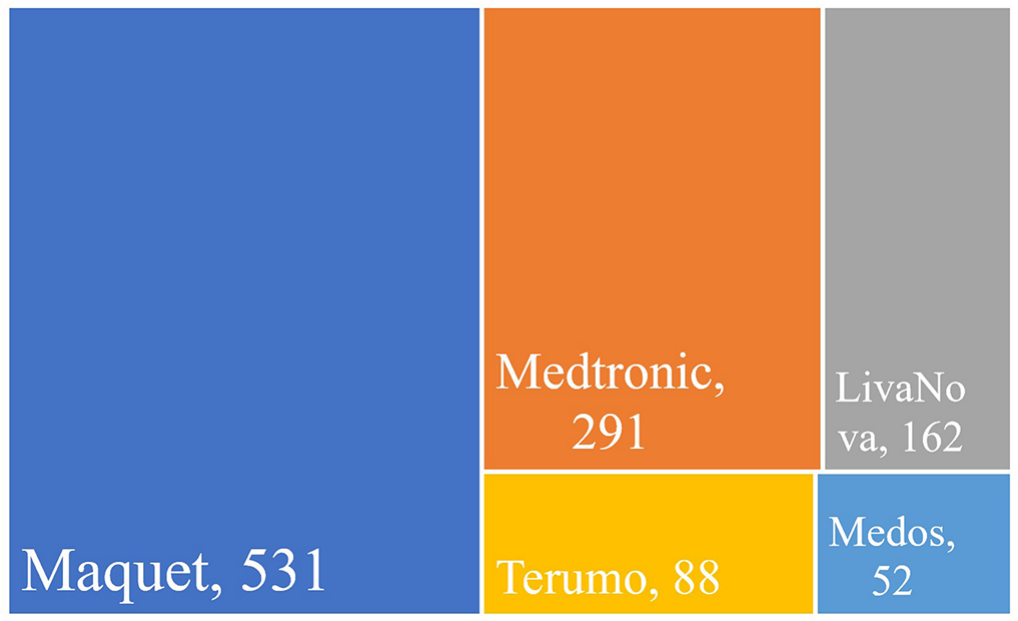
Number of research papers using different branded products in the field of ECMO, 2018–2022.

An analysis of ECMO-related research papers revealed that 71% of the papers were concentrated in journals owned by five publishing groups: Elsevier, Lippincott Williams & Wilkins, Sage, Springer Nature, and Wiley. The number of ECMO research papers related to major brands from 2018 to 2022 was analyzed to reveal the market share of major brands while the document type was refined as “article” (Figure 7).

Maquet corporation (Germany) was the market leader of ECMO (33), with 531 papers using Maquet products from 2018 to 2022, far more than the other four brands. Medtronic (United States) (34) and LivaNova (UK) (35) followed in order, with 291 and 162 papers, respectively. Maquet, Medtronic, and LivaNova were the top three ECMO providers worldwide, occupying a major market share. There were also 88 papers from Terumo (Japan) (36) and 52 papers from Medos (Germany) (37).

## 4. Discussion

ECMO, as an extracorporeal life support system, provides respiratory and hemodynamic functional support with an artificial heart pump and artificial membrane lung. It can alleviate circulatory failure and intractable hypoxemia in patients with cardiopulmonary failure, gaining time for the treatment of the primary disease and recovery of cardiopulmonary function. In the past 50 years, ECMO has been widely used in neonatal, pediatric, and adult care, while its application scenarios included respiratory diseases, circulatory failure, pre-hospital emergency, emergency medicine, critical care medicine, bridging organ transplant, donor organ function protection, inter-hospital transfer, war wound treatment, and many other fields. The COVID-19 outbreak in late 2019 posed a significant threat to global public health security. ECMO can temporarily replace and support the cardiopulmonary function of critical patients with COVID-19. It is a key medical device urgently needed in the current epidemic prevention and control work. Practitioners in the ECMO industry have conducted a lot of clinical and basic research to clarify its indications and contraindications, control its complications, expand its application scenarios, improve its clinical treatment specifications and quality control system, and optimize its team management and training programs to achieve more satisfactory treatment results, rapidly making ECMO technology mature in clinical practice. However, the high technical threshold, high risk, and high cost of ECMO itself still greatly restrict its promotion and popularization, and further optimization and improvement are urgently needed.

Bibliometrics is an interdisciplinary science that can effectively identify development trends, summarize research hotspots, and predict research topic directions in an era of a great abundance of literature research, providing key technical support for practitioners and researchers in the ECMO industry. Based on bibliometric expertise, we summarized and analyzed the development trends and industry hotspots of ECMO technology, expecting to further promote the development of ECMO technology in P.R.China.

From the keyword co-occurrence clustering analysis of ECMO research papers, ECMO has been widely used for years to treat cardiopulmonary diseases that are difficult to improve with conventional treatment and has been widely used in the fields of short-term assistance after surgery, organ transplantation, critical care medicine, biomedical engineering, and pediatrics. After the analysis of specific study topics, it was seen that VA ECMO had been the most studied in cardiogenic shock, cardiac arrest, and ECPR, especially in postoperative cardiac circulatory support, pulmonary embolism, and emergency ECMO rescue in acute infarction, to improve patient prognostic outcomes and complication management for further in-depth study. The main battlefield of VV ECMO is still in ARDS for achieving adequate oxygenation and carbon dioxide removal. Therefore, research on the role of VV ECMO in the fight against the COVID-19 pandemic is of interest. Additionally, some large clinical centers in China have accumulated rich experience in ECMO-assisted treatment of adult ARDS patients, lung transplantation, and the field of intra- and inter-hospital long-distance transport.

Although the role of ECMO-assisted technology in the main battlefield of cardiopulmonary circulatory failure treatment is gradually becoming widely recognized, the prevention, early diagnosis, and treatment of its related complications have been the focus and difficulty of researchers. We will continue to explore these research hotspots in the future. At this stage, complications of ECMO are usually classified into two main categories, namely, patient organism complications (bleeding, embolism, infection, renal failure, neurological injury, and limb ischemia) and ECMO technology-related complications (oxygenator dysfunction, intubation-related complications, oxygenator or line thrombosis, tube dislodgement or rupture, manual pump mechanical or control failure, and heat exchanger failure). Complications associated with the ECMO technique are most common with oxygenator dysfunction and thrombosis. With the application of polymethylpentene (PMP) hollow fiber membrane oxygenators, innovations in pipeline anticoagulation coating, and optimization of magnetic levitation centrifugal pumps, the incidence of mechanical failures mentioned above has been greatly reduced. Among the patients' complications, dyscoagulation, infection, and neurological disorders are the most relevant to morbidity and mortality rates and prognosis of ECMO-assisted patients, which still require multidisciplinary and interdisciplinary cooperation. Therefore, as a high-consumption advanced life support technology, ECMO is accompanied by various potential complication possibilities in enhancing patient survival. It is necessary to start from many aspects, such as developing and improving the performance of core ECMO components and optimizing intubation techniques, selecting the optimal timing of ECMO application for different indications, reasonably grasping the indications and timing of ECMO transfer and shutdown, optimizing ECMO operation monitoring, and strengthening ECMO team building and technical training, for ECMO technology to maximize patient benefits.

Improving portability and mobility will also become a hot research and development trend for ECMO (38). This has important clinical value for performing ECPR in patients with in-hospital or out-of-hospital cardiac arrest, as well as for ECMO-assisted inter-hospital transfer of patients over medium and long distances, or even for long-distance evacuation of combat casualties with heart and lung failure at the scene of war. There are reports of successful use of Cardiohelp, a portable ECMO device, for ECMO medical teams to treat patients with severe ARDS and achieve intercontinental transfers with satisfactory prognosis (38, 39).

The COVID-19 pandemic further highlights the clinical significance and market value of ECMO. As the cost of ECMO treatment remains high, the most used ECMO product giants are concentrated in few European and American companies, such as Maquet, Medtronic, and LivaNova. For the time being, there are no domestic manufacturers of ECMO in China. Realizing the localization of ECMO core components is the key point to breaking through the price and technical barriers. The core of the new generation of the artificial blood pump is the design of magnetic levitation technology and a high-efficiency impeller, and the key point is to improve the pumping efficiency and blood compatibility. The core material of the oxygenator, PMP hollow fiber membranes, is available from only one supplier in the world, 3 M. At present, few teams from domestic Chinese institutions have made a big breakthrough in the field of localization of ECMO devices, and there are successful cases of domestic ECMO being applied to clinical patients. The teams include MicroPort, Chinabridge (Shenzhen) Medical, Jiangsu STMed, Magassist, China Aerospace, and other companies, as well as Tsinghua University, Shandong University, and Xi’an Jiaotong University, in conjunction with the National Engineering Research Center for Biomaterials (NERCB) at Sichuan University. The localization of ECMO core equipment is crucial to reduce the cost of use, improve patient acceptance, and promote the inclusion of national medical insurance support. We expect that the breakthrough in the field of localization can further promote the reduction of ECMO treatment costs and improve the acceptance of the general public and the popularity of regional central hospitals; thus, more patients can benefit from ECMO.

There are also some limitations in this paper. First, we did not distinguish between original R&D literature, clinical application literature, and technical method improvement literature. Hence, the evaluation of original R&D literature with a higher academic value might have been biased. Second, this article was an analysis of ECMO R&D trends based on literature research without further analysis of core patent applications and layouts for membrane lung, centrifugal pump, and anticoagulation coating. It had limited significance for guiding the R&D of ECMO core components, and we will conduct comprehensive research on ECMO core technology patents in subsequent articles.

Using scientometric methods and visualization software, this study provided an overview of the progress of ECMO global research activities, industry layout, and hotspot analysis since the inception of extracorporeal circulation technology in the 1950 s and analyzed the current stage of ECMO development trends. Although ECMO technology has been widely performed, as an invasive, high-risk, and high-consumption medical technology, further clinical studies are still needed to maximize the clinical benefit population and get the best balance of risk and benefit for specific patients. As for the road of ECMO localization, it is also necessary to make good use of the “latecomer advantage” and continue to make breakthroughs in the fields of biocompatibility, monitoring intelligence, and portable integration of equipment and consumables to promote the realization of completely independent intellectual property rights of ECMO equipment and consumables. The highest goal of ECMO research and technology application is to better serve the majority of patients and allow them to benefit from ECMO.

## Data Availability

The raw data supporting the conclusions of this article will be made available by the authors, without undue reservation.
