# Supplementary material for: Research status and development trend of extracorporeal membrane oxygenation based on bibliometrics
Source: Front Cardiovasc Med. 2023 Mar 10;10:1048903. doi: 10.3389/fcvm.2023.1048903 (PMC10036781; doi:10.3389/fcvm.2023.1048903)
Supplement: Supplementary file 1 [file RetrievalstrategyofECMO-relatedscientificpublications.docx]

**Retrieval strategy of ECMO-related scientific publications**

This study used the Science Citation Index Expanded (SCIE) database as the source of data collection, and the data were searched in the time range up to December 31, 2022. The search time was January 25, 2023. All papers related to ECMO research were searched by title, keywords and topic, and the valid papers included 9082 papers after refining the document type as “article”. Review articles are not included in this analysis to avoid slanting the results in one direction or another.


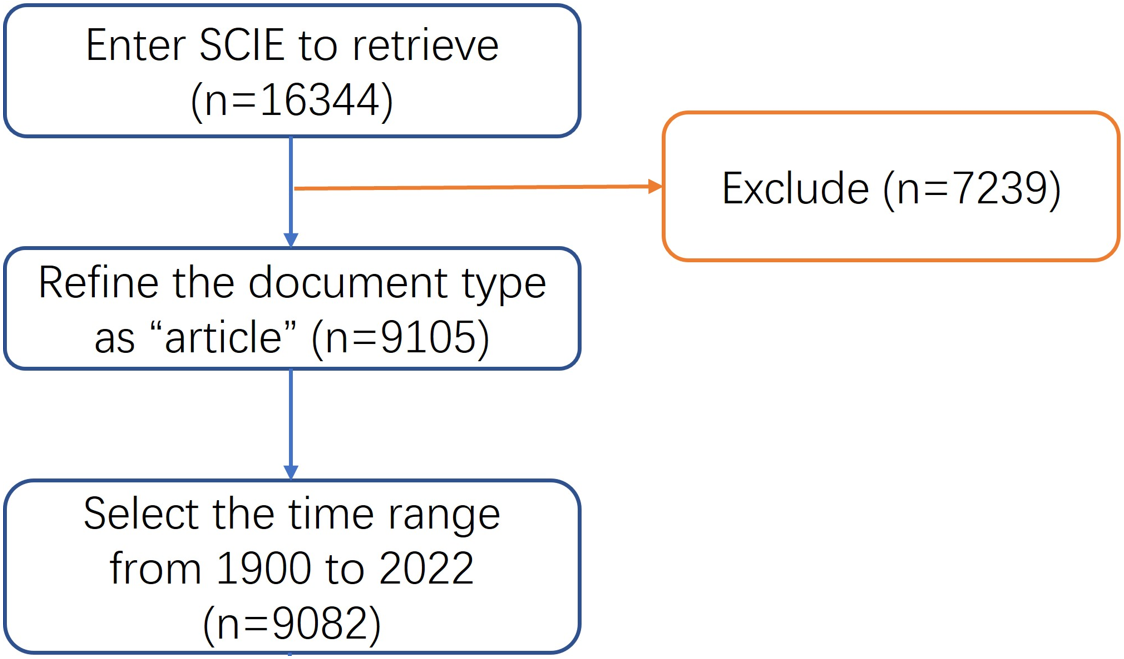


Query in SCIE database:

(ti=(ECMO or “extra corporeal membrane oxygenation” or “extracorporeal membrane oxygenation”) or ak=(ECMO or “extra corporeal membrane oxygenation” or “extracorporeal membrane oxygenation”)) or (ti=(ECLS or “extracorporeal life support” or ECLA or “extracorporeal lung assist”) and ts=((“ECMO” or “extra corporeal membrane oxygenation” or “extracorporeal membrane oxygenation” or “membrane lung” or “artificial lung” or “artificial pump-lung” or “membrane oxygenator” or “membrane type oxygenator” or “cardiopulmonary bypass”) not (ozone OR electro-catal* OR electrocatal* OR electrochem*))) or (ti=(“membrane lung” or “artificial lung” or “artificial pump-lung” or “membrane oxygenator” or “membrane type oxygenator”) and ts=((membrane) not (ozone or electro-catal* or electrocatal* or electrochem*))) or (ti=(oxygenator) and ts=((ECMO or “extra corporeal membrane oxygenation” or “extracorporeal membrane oxygenation” or “membrane lung” or “artificial lung” or “artificial pump-lung” or “membrane oxygenator” or “membrane type oxygenator” or “cardiopulmonary bypass” or “extracorporal circulation” or “extracorporeal blood circuit” or “extracorporeal blood circulation” or “extra corporeal blood circulation” or “extracorporeal circulation” or “extracorporeal circulation of blood” or ECLS or “extracorporeal life support” or ECLA or “extracorporeal lung assist”) not (ozone or electro-catal* or electrocatal* or electrochem*))) or (ti=(“blood pump*”) and ts=((ECMO or “extra corporeal membrane oxygenation” or “extracorporeal membrane oxygenation” or “membrane lung” or “artificial lung” or “artificial pump-lung” or “membrane oxygenator” or “membrane type oxygenator” or “cardiopulmonary bypass” or ECLS or “extracorporeal life support” or ECLA or “extracorporeal lung assist”) not (ozone or electro-catal* or electrocatal* or electrochem*))) or ((ts=((“extracorporeal membrane oxygenation” or “extra corporeal membrane oxygenation” or ECMO or miniECMO or ((extracorporeal or “extra corporeal”) and “gas exchange”) or ((extracorporeal or “extra corporeal”) and blood and oxygenat* and membrane*) or “artificial lung” or “extracorporeal lung” or “extracorporeal ambulatory lung” or “artificial heart lung machine” or “artificial pump lung” or “membrane lung” or oxygenator* or “extracorporeal life support” or ECLS or “extracorporeal lung assist*”) and membrane* and (coat* or biocoat* or cover* or “medicine film” or “surface modification” or “poly(ethylene oxide)” or “poly(2-methoxyethyl acrylate)” or “poly2-methoxyethylacrylate” or PMEA or “phosphoryl choline” or phosphorylcholine or “polyethylene glycol” or “poly(ethylene glycol)” or carmeda or trillium or bioline or safeline) not (ozone or electro-catal* or electrocatal* or “electro catalysis” or electrochem* or electro-chem*)) not ti=(“bioartificial liver*” or “artificial liver*”)) and ts=(hydrophilic* or hydrophobic or biocompatib* or hemocompatib* or hemo-compatible or anticoagulat* or anticoagulant or antithrombotic or anti-coagulant or anti-coagulat* or anti-thrombotic or “blood compatible” or “blood compatibility” or “organism compatible” or thromboresist* or “bioactive coating”)) or ((ti=(“centrifugal pump*”) or ak=(“centrifugal pump*”)) and ts=(blood*))
